# Low hepcidin in liver fibrosis and cirrhosis; a tale of progressive disorder and a case for a new biochemical marker

**DOI:** 10.1186/s10020-018-0008-7

**Published:** 2018-03-15

**Authors:** Driton Vela

**Affiliations:** Department of Physiology, Faculty of Medicine, University of Prishtina, Martyr’s Boulevard n.n, Prishtina, 10000 Kosovo

**Keywords:** Alcohol, HCV, Hepcidin, liver fibrosis

## Abstract

Liver fibrosis is a precursor of liver cirrhosis, which is associated with increased mortality. Though liver biopsy remains the gold standard for the diagnosis of fibrosis, noninvasive biochemical methods are cost-effective, practical and are not linked with major risks of complications. In this respect, serum hepcidin, has emerged as a new marker of fibrosis and cirrhosis. In this review the discussion uncovers molecular links between hepcidin disturbance and liver fibrosis/cirrhosis. The discussion also expands on clinical studies that suggest that hepcidin can potentially be used as a biochemical parameter of fibrosis/cirrhosis and target of therapeutic strategies to treat liver diseases. The debatable issues such as the complicated nature of hepcidin disturbance in non-alcoholic liver disease, serum levels of hepcidin in acute hepatitis C virus infection, cause of hepcidin disturbance in autoimmune hepatitis and hepatic insulin resistance are discussed, with potential solutions unveiled in order to be studied by future research.

## Background

**Fig. 1:**
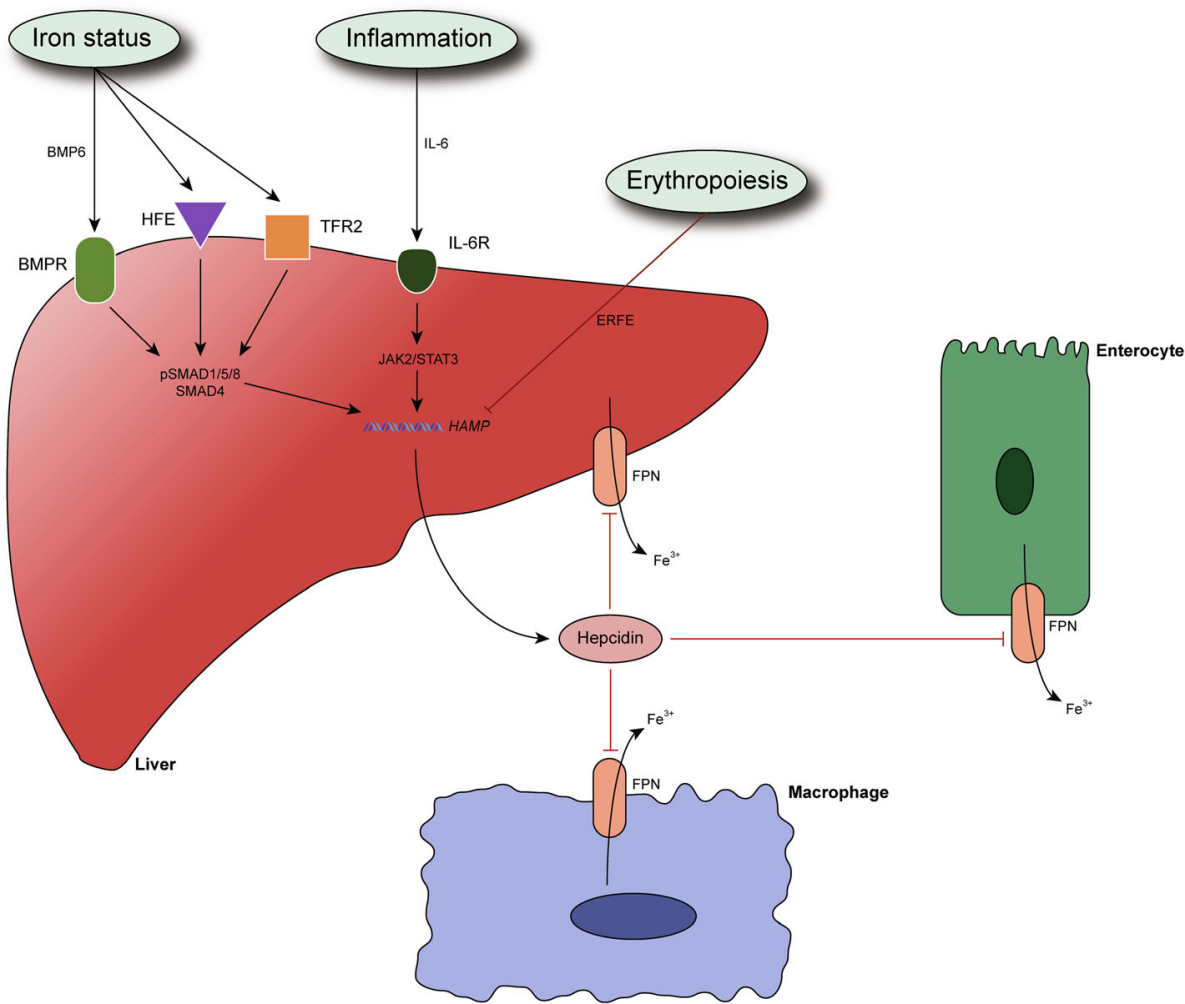
Hepcidin expression and mode of action. BMP6, bone morphogenetic protein 6; BMPR, BMP receptor; ERFE, erythroferrone; Fe, iron; FPN, ferroportin; HAMP, hepcidin antimicrobial peptide; HFE, hemochromatosis protein; IL-6, interleukin 6; IL-6R, IL-6 receptor; JAK2, janus kinase 2; SMAD proteins, s-mothers against decapentaplegic proteins; STAT3, signal transducer and activator of transcription 3; TFR2, transferrin receptor 2. Hepcidin expression is primary regulated by iron-status, inflammation and erythropoietic drive. Iron-status induces hepcidin expression by through BMPR ligands such as BMP6. BMPR activates SMAD pathway which increases hepcidin expression through HAMP transcription. Inflammation induces hepcidin expression through cytokines like IL-6 which activates JAK2/STAT3 pathway in heptocytes. This pathway increases hepcidin expression. Erythropoiesis suppresses hepcidin expression probably through erythroferrone produced by erythrocyte precursors. Once released by hepatocytes in plasma, hepcidin reaches target cells like enterocytes and macrophages, where hepcidin induces FPN degradation. This action reduces iron efflux from cells, because FPN is the major exporter of iron out of cells

Hepcidin is an ubiquitous antimicrobial peptide found in different species, including humans (Segat et al. 2008). Though initially discovered for its antimicrobial properties, in 2001 scientists found that, in fact, hepcidin is the major regulator of iron metabolism (Ganz 2011; Nicolas et al. 2001). Since then we have learned that hepcidin is mostly produced by hepatocytes in response to iron-load in cells. Whenever this load increases, hepcidin expression goes up in hepatocytes, which results in increased serum hepcidin levels (Nicolas et al. 2001). Hepcidin main mode of action is realized through its binding with ferroportin (FPN) in target cells, like enterocytes, macrophages, hepatocytes (Fig. 1). FPN is the major protein channel that regulates iron export from cells. Its complex with hepcidin induces FPN degradation inside cells (Nemeth et al. 2004). This means that high levels of hepcidin reduce the levels of iron in serum. In this way hepcidin protects us from iron-overload. This role of hepcidin is important since there is no known excretory pathway for body iron. Keeping iron in check through hepcidin is vital for our cells, because high levels of iron saturate the capacity of the proteins to keep iron in bound form (Loréal et al. 2000). Excess iron can cause oxidative damage, but also can be used by microbes to maintain their survival (Puntarulo 2005; Skaar 2010). This is the reason why infection with bacteria can cause increased mortality in patients with high iron-overload (Skaar 2010). On the other hand, low levels of iron cause anemia, but paradoxically whenever the immune system of patients is damaged in chronic diseases (autoimmune and inflammatory) our organism sets “priorities” by choosing anemia as the “norm” in these situations, which seems to protect us from potentially dangerous infections (Zarychanski and Houston 2008; Roy 2010). In this everlasting fight of our cells to use iron efficiently and as discreetly as possible, maintaining hepcidin balance is crucial to prevent disease and organ damage.

## Hepcidin production in liver

Hepatocytes express 15-1500 times more hepcidin than other cells in the body, thus making them the primary source of hepcidin (Krause et al. 2000). This role is perfectly suited for hepatocytes, since they are exposed to the iron absorbed from enterocytes and iron released from macrophages through portal circulation. In basal conditions hepcidin expression is controlled through iron-load. Iron-load stimulates production of bone morphogenetic protein 6 (BMP6). BMP6 creates a complex with BMP receptor (BMPR) which in turn increases hepcidin expression through intracellular s-mothers against decapentaplegic (SMAD) pathway (Babitt et al. 2006; Steinbicker et al. 2011; Wang et al. 2005; Kautz et al. 2008). The source of BMP6 in liver are liver sinusoidal endothelial cells (LSEC) (Rausa et al. 2015; Canali et al. 2017). This role suits LSEC because of their direct contact with plasma and their intimate relationship with hepatocytes. LSEC are known for their high endocytic activity, which makes them ideal cells for “extracting” plasma iron transporters such as transferrin or serum ferritin (Maslak et al. 2015; Feng et al. 2012; Parrow and Fleming 2017). Experimental inactivation of BMP6 causes serious iron-overload, while recent evidence suggests that BMP6 mutations could be the source of a mild but still unrecognized form of hemochromatosis (HH) (Daher et al. 2016; Piubelli et al. 2017). Less is known about the mechanism by which nonparenchymal liver cells secrete BMP6. Ferritin has been proposed as a potential sensor of iron (Feng et al. 2012), but more studies should explore this possibility, as well as other potential molecules.

Acute and chronic iron-load exert their control on hepcidin expression by partially independent mechanisms. This is enforced by observations in BMP6 and hemojuvelin (HJV) knockout models in mice, where chronic iron-load does still induce hepcidin expression (albeit, in a blunted manner) by an unknown route (Ramos et al. 2011).

Other factors that control hepcidin expression include erythropoietic drive, hypoxia and inflammation, serving as powerful inducers (inflammation) and suppressors (erythropoiesis, hypoxia) of hepcidin expression, often by overrunning the major BMP6 route of hepcidin regulation (Nemeth et al. 2004; Pak et al. 2006; Piperno et al. 2011). In specific situations these factors increase iron availability or reduce iron-load depending on the needs of our cells. For example, during inflammation hepcidin expression is upregulated, while iron levels start to drop as a consequence. During infections this response is beneficial, because it serves as a protective mechanism against extracellular microbes. This is enforced by studies where supplementation with hepcidin analogs protects from infections (Paradkar et al. 2008; Michels et al. 2017). Inflammation induces hepcidin expression through janus kinase/signal transducer and activator of transcription (JAK/STAT) pathway, though SMAD pathway has been shown to be affected during inflammation as well (Wrighting and Andrews 2006; Canali et al. 2016). Recent evidence suggests that SMAD pathway activation during inflammation could occur in nonparenchymal liver cells and it is not correlated to hepcidin expression (Besson-Fournier et al. 2017).

## Mechanisms of low hepcidin in liver disease

**Fig. 2:**
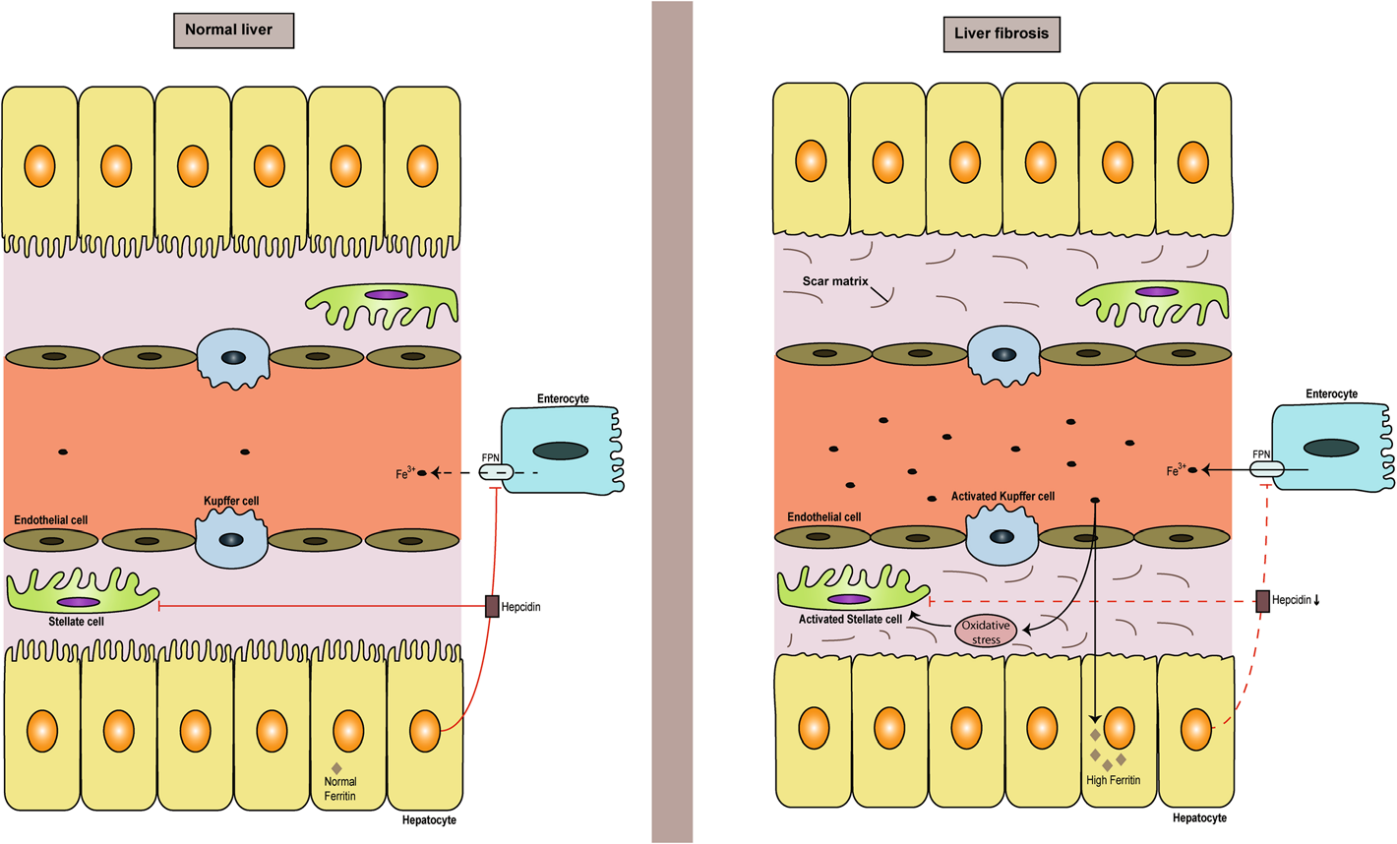
Hepcidin (in)actions in normal liver and liver fibrosis. Fe, iron; FPN, ferroportin. In normal liver, hepcidin produced by heptocytes controls iron levels in plasma by preventing excessive iron absorption from enterocytes. In this way hepcidin protects liver form iron-load. But hepcidin can protect liver by inactivating hepatic stellate cells as well. In liver fibrosis low hepcidin causes high iron-load and oxidative stress. Oxidative stress and lack of hepcidin-induced supression of hepatic stellate cells induces their activation, which results in deposition of scar tissue and liver fibrosis

As we have previously mentioned, hepcidin is a peptide which is under strict control of different regulatory mechanisms. These mechanisms can become dysregulated and cause inappropriate levels of hepcidin. In this respect chronic low levels of hepcidin are of interest for researchers, because low hepcidin can cause iron overload and increased oxidative stress in liver (Nicolas et al. 2001; Puntarulo 2005) (Fig. 2). Increased oxidative stress in combination with other factors (genetic, viruses, alcohol, autoimmune process, hepatotoxins, diet, nonalcoholic steatohepatitis) can result in liver fibrosis (Bataller et al. 2005). Liver fibrosis is the consequence of chronic liver damage, characterized by increased deposition of extracellular matrix induced by activated hepatic stellate cells (HSCs), which promotes creation of fibrous scars in the liver. This fibrotic tissue can eventually reorganize and disrupt liver architecture, by creating regenerative nodules, which is the main feature of the end-damage caused by the scaring process, that is, liver cirrhosis (Bataller et al. 2005).

In liver disease, low hepcidin is linked with many conditions, but the mechanisms behind low levels of hepcidin are still elusive and remain to be fully explained. In next paragraphs the discussion attempts to unveil what is known about hepcidin dysregulation in different liver diseases.

### Low hepcidin in HH

Loss of hepcidin signaling and hepcidin expression is the pathogenic mechanism behind one of the most prevalent genetic diseases in Europe (HH). The mechanisms behind low levels of hepcidin in this disease are known and they include defective signaling during hepcidin expression; through HFE (most common type) in HH type 1 or HJV in HH type 2A, or transferrin receptor 2 (TFR2) in HH type 3, or by direct mutations in hepcidin antimicrobial peptide (*HAMP)* gene in HH type 2B. There is also HH type 4, characterized by defective FPN or by defective hepcidin action on FPN (Pietrangelo 2010).

Iron-load in HH is higher in patients with liver fibrosis than those without (Loréal et al. 1992), which suggests a cause/effect relationship between significant iron-load and liver fibrosis in HH. Still, we have to keep in mind that in HH, liver fibrosis is not present in most patients, which means that mild iron-load is not a great risk factor in liver fibrosis. Also, liver cirrhosis as the final progression of liver fibrosis, is rarely encountered in patients with ferritin values less than 1000 μg/L (Valenti et al. 2010; Schöniger-Hekele et al. 2002; Bassett et al. 1986; De Gobbi et al. 2002). Decades of study have acknowledged that HH is not one disease but a term that encompasses many diseases characterized with different aberrations in hepcidin expression and function. As a result, we have e clearer picture about the differences in the level of liver damage and prevalence of liver fibrosis in different forms of HH. In HH type 1 significant iron-load is evidenced relatively rarely (Valenti et al. 2010), but in HH due to mutations in HJV and *HAMP* gene the level of iron-load is higher, the clinical presentation more severe and the parenchymal damage is present in early life (De Gobbi et al. 2002). This is not much of a surprise since HFE is not a powerful inducer of hepcidin expression. In HH-HFE clinical penetrance is related to male sex, alcohol consumption, viral hepatitis (Alexander and Kowdley 2009). Furthermore, the presence of *HFE* mutations is not significantly related to the severity of liver fibrosis (Valenti et al. 2010).

### Low hepcidin in alcoholic liver disease (ALD)

Alcohol is an already established inducer of hepatocyte damage, which can progress to overt liver fibrosis. Suspected mechanisms of alcohol-induced liver fibrosis include increased levels of lipopolysaccharide (LPS), activation of HSCs and inhibition of antifibrotic actions (Gao and Bataller 2011).

**Fig. 3:**
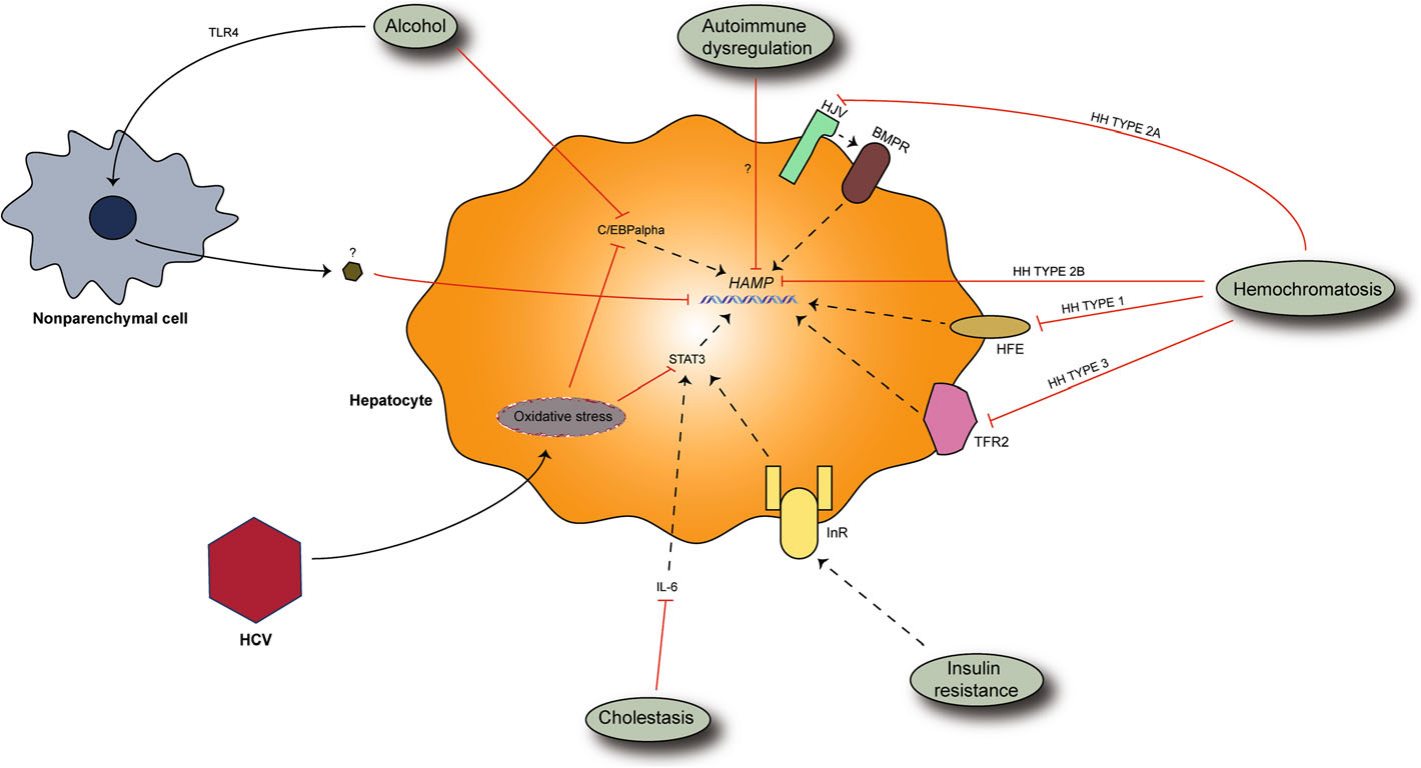
Mechanisms behind low levels of hepcidin in liver disease. BMPR, bone morphogenetic protein receptor; C/EBP alpha, CCAAT enhancer binding protein alpha; HAMP, hepcidin antimicrobial peptide; HCV, hepatitis C virus; HFE, hemochromatosis protein; HH, hemochromatosis; HJV, hemojuvelin; IL-6, interleukin 6; InR, insulin receptor; STAT3, signal transducer and activator of transcription 3; TFR2, transferrin receptor 2; TLR4, toll-like receptor 4. Different pathogenic factors are responsible for low values of hepcidin. In HH mechanisms behind low levels of hepcidin are already known and they include defective signalling though HJV, HFE, TFR2 or through direct inhibition of HAMP gene. Alcohol inhibits hepcidin expression through its actions on C/EBP, but also indirectly through TLR4 pathway. It is believed that TLR4 mediates this action of alcohol through nonparenchymal liver cells, but the paracrine signal responsible for this effect is unknown. HCV also suppresses hepcidin expression through oxidative stress, which inhibits C/EBP and STAT3 actions on HAMP. Cholestasis, on the other hand, suppresses hepcidin expression by inhibiting IL-6/STAT3 pathway. In AILD mechanisms behind low levels of hepcidin are unknown. In hepatic insulin resistance defective insulin signaling is linked with defective hepcidin expression, partially through STAT3 pathway

Alcohol is also linked with disturbances in levels of hepcidin. It is interesting to notice that, in alcoholic patients, low levels of hepcidin are observed even with preserved liver function (Costa-Matos et al. 2012). This would suggest that alcohol is a primary cause of low levels of hepcidin, and not a consequence of alcohol-induced liver damage. The rationale behind this observation is the direct effect of alcohol on hepcidin expression. Alcohol can inhibit hepcidin expression through its suppressive effects on CCAAT-enhancer-binding protein (C/EBP) in hepatocytes, at the same time counteracting iron-induced activity of this transcription factor, thus rendering iron-induced hepcidin expression ineffective (Harrison-Findik et al. 2007) (Fig. 3). In addition, the upregulation of divalent metal transporter 1 (DMT1) and FPN in enterocytes increases serum iron levels and cellular iron-load, which is linked with liver fibrosis (Harrison-Findik et al. 2007). This effect of alcohol can be reversed with treatment by antioxidants, which is not surprising since alcohol induces oxidative stress (Harrison-Findik et al. 2006). This is the reason why progression rate of fibrosis is twice as high in steatotic drinkers compared to steatotic nondrinkers (Serfaty et al. 2002). Another mechanism of hepcidin suppression by alcohol includes suppression through toll-like receptor 4 (TLR4) pathway. TLR4 is a transmembrane protein involved in innate immune responses. In mice with defective TLR4 receptor alcohol cannot suppress hepcidin expression (Zmijewski et al. 2014). It is interesting to notice that TLR4 deficiency protects from liver fibrosis, making it an interesting candidate to be studied in the context of alcohol-induced hepcidin down-regulation (Weber et al. 2016; Seki et al. 2007). The mediator cell of TLR4 signaling remains to be found, and it seems that Kupffer cells are not involved in alcohol-induced hepcidin expression (Harrison-Findik et al. 2008). Hepatocytes are unlikely candidates as well, since their expression of TLRs is low, while their reaction to TLR ligands is weak. A plausible candidate seems to be HSCs, since they express different TLRs and react in response to their actions (Yang and Seki 2012).

Alcohol might disrupt canonical hepcidin pathways such as BMPR/SMAD pathway, but also can suppress hepcidin via hypoxic signals, though the importance of these alcohol-induced actions on hepcidin expression remain to be confirmed (Gerjevic et al. 2012; Heritage et al. 2009).

It seems that alcohol consumption in the setting of iron-overload can serve as a strong inducer of liver fibrosis. In HH patients, alcohol consumption of more than 60 g/day increases the risk of cirrhosis by 9 fold (Fletcher and Powell 2003). This increase in risk of progressive liver damage in alcoholic HH patients is in-line with the so-called “multiple hit” scenario, where two or more pathophysiological factors induce hepatocyte damage in a complementary manner, which eventually leads to liver fibrosis (Takaki et al. 2013). There are other similar examples of “multiple hit” factors adding to the liver damage; in mice fed with long term high cholesterol diet and alcohol, liver shows signs of early fibrosis, compared to individual effects of high cholesterol and alcohol (Krishnasamy et al. 2016). The risk of liver cirrhosis is dramatically increased in patients with hepatitis C virus (HCV) infection, when HCV infection is accompanied with heavy alcohol consumption (Harris et al. 2001).

Recently, the effect of alcohol in hepcidin has been suggested to be more complex than previously thought (Harrison-Findik and Lu 2015). Also, alcohol might cause a reduction in hepcidin values through ubiquitous proteins involved in liver regeneration, but the importance of this finding remains to be evaluated (Kumar et al. 2016).

As we can see the evidence for alcohol-induced low levels of hepcidin is considerable and some of the mechanisms behind these disturbances in hepcidin levels have already been elucidated, but the full picture remains to be solved.

### Low hepcidin in HBV- and HCV-induced liver disease

Infection with hepatitis B virus (HBV) and HCV is a known causative factor in liver fibrosis (Ohkoshi et al. 2016; Bataller et al. 2004).

The mechanisms behind HCV-induced liver fibrosis include induction of reactive oxygen species (ROS) which impairs C/EBP and STAT3 binding to hepcidin promoter (Miura et al. 2008). Low levels of hepcidin in HCV infection deteriorate liver function by serving as a strong factor in inducing liver fibrosis (Angelucci et al. 2002; Chapoutot et al. 2000; Horl and Schmidt 2014). It has been suggested that by lowering hepcidin, HCV protects itself from antiviral innate immune responses, since hepcidin can inhibit HCV replication (Liu et al. 2012). In chronic HCV infection, patients mRNA of liver hepcidin is correlated with iron status and not with virus load or fibrosis stage (Aoki et al. 2005). This would suggest that hepcidin regulation is preserved in response to iron status. Low hepcidin in HCV is more clearly linked with end-stage liver disease than with early fibrosis (Nagashima et al. 2006). Data suggest that though hepcidin expression in chronic HCV infection is induced by iron status, this upregulation is not sufficient, which is consistent with the finding of the impaired binding of hepcidin upregulators to *HAMP* seen in HCV infection due to increased oxidative stress (Miura et al. 2008; Fujita et al. 2007). What is more, HCV is known to induce more pronounced oxidative stress than other viruses that cause hepatitis (Valgimigli et al. 2000; Farinati et al. 1995). Consolidating this argument is the success seen during treatment of HCV infection with antiviral therapy which restores hepcidin levels by increasing the expression of STAT3 (Ryan et al. 2012), while keeping in mind that loss of STAT3 is linked with increased susceptibility to oxidative stress (Barry et al. 2009). Iron reduction that ensues after antiviral therapy is associated with reduced viral load. It has to be noted that phlebotomy reduces iron-load and markers of oxidative stress which is why long-term treatment with phlebotomy improves liver histology, but also reduces the risk of progression to hepatocellular carcinoma (Kato et al. 2001; Yano et al. 2002; Kato et al. 2007). But, according to one study, phlebotomy does not correct the inappropriate hepcidin response to ferritin load (Sugimoto et al. 2009). This might be explained by differences in short-term vs long-term treatment success, but also by timing of the interventions, which means that phlebotomy in advanced liver disease induced by HCV might not recuperate hepcidin expression, though this hypothesis need to be evaluated by further studies.

Hepcidin expression during HCV infection is complicated by the effect of genetic factors. These factors have been shown to predispose patients with HCV to a different treatment potential of anti-HCV drugs (Wróblewska et al. 2017).

According to a paper from Foka et al., HCV in acute setting upregulates hepcidin expression to increase iron availability for viral replication, while in chronic setting hepcidin levels go down, but do get increased in response to viral load (Foka et al. 2016). But, this and other in-vitro studies are in contradiction with other studies where hepcidin is clearly low in HCV replicon and infected cell lines (Miura et al. 2008; Liu et al. 2012; Bartolomei et al. 2011). These contradictions might have occurred because of differences in models of study. Clinical data suggest that hepcidin expression is unchanged or goes down in acute HCV infection during peak viremia, but this observation should be validated by larger studies (Armitage et al. 2014). Similarly, Foka et al. results from their clinical study confirm that hepcidin levels go down in chronic HCV infection, while results from patients with acute HCV infection showed increased levels of hepcidin, which was also observed in chronic HCV patients with high viremic load. Acutely infected HCV patients were all males (compared to other groups) which could affect the reliability of hepcidin results. Hepcidin levels show gender differences and they should be taken into account to avoid false results (Galesloot et al. 2011). At the same time, the group of patients with acute HCV infection showed nearly double the levels of ferritin compared to other groups, which could mean that increased levels of hepcidin in this group was a result of reactive response to increased iron-load. Unfortunately, this study did not provide detailed correlation reports between the examined variables. Still, this would not explain the differences seen between chronic HCV patients with low and high viral load. On closer look average hepcidin values between these groups did not show great differences compared to more prominent increased levels in acute HCV infection. Also, we have to be careful when examining differences in hepcidin levels in such a small group of patients because small differences in levels of hepcidin might be e result of chance and could reflect variations in normal range values.

In chronic HBV infection levels of hepcidin also change; they rise in early stages of the disease, only to be reduced in the cirrhotic stage of the disease, reflecting the inability of hepatocytes to control hepcidin levels caused by destruction of liver architecture during fibrosis (Wang et al. 2016; Lin et al. 2013). Differences in pathophysiological mechanisms induced by HBV and HCV explain why hepcidin levels have a specific mode of fluctuation in these infections; they include differences in the level of oxidative stress, co-infection with hepatitis D, level of viral load, presence of inflammation (Aoki et al. 2005; Fujita et al. 2008; Sebastiani et al. 2012; Wang et al. 2013). On the other hand, HBV mode of action in the liver is to evade innate immune recognition, while HCV counteracts the already activated immune response. Therefore, low hepcidin expression could be a defensive strategy of HCV through which HCV counteracts hepcidin role in suppressing HCV replication (Liu et al. 2012; Wieland and Chisari 2005).

### Low hepcidin in autoimmune liver disease (AILD)

Levels of hepcidin are low in newly diagnosed patients with liver autoimmune disease (Lyberopoulou et al. 2015; Tan et al. 2012). The reason behind this observation remains a mystery, but it is pertinent to speculate that the immune system disturbances that cause the liver disease are behind low levels of hepcidin.

In mouse models of autoimmune diabetes, the autoimmune process can be dampened by beta-cells of the pancreas by inducing cathelin-related antimicrobial peptide (CRAMP) expression, which is an antimicrobial peptide “cousin” to hepcidin (Sun et al. 2015; Kościuczuk et al. 2012). This suppression of autoimmunity by beta-cells of the pancreas is mediated through gut microbiota signaling, which is an often overlooked factor in hepcidin expression (Shanmugam et al. 2014). Similarly, it might be that the autoimmune process in the liver could disrupt hepcidin expression due to dysregulation of immune response. This hypothesis would mean that hepcidin has a role in immune responses, similar to other antimicrobial peptides, like the aforementioned CRAMP. Other circumstantial evidence might help us understand disturbances of hepcidin levels in AILD; IL-22 has been shown to induce hepcidin expression during its control of early immune response (Armitage et al. 2011), while disruption of this cytokine in T-cell mediated hepatitis causes progressive damage in the liver (Pan et al. 2014). In conclusion, data from studies on the role of autoimmune process in AILD in lowering hepcidin expression are sparse, and remain to be studied by future research.

### Low hepcidin in other liver diseases

Hepcidin disturbance has been observed in a range of liver diseases. In one of the most prevalent liver disease named non-alcoholic fatty liver disease (NAFLD) levels of hepcidin are higher compared to control (Senates et al. 2011; Vuppalanchi et al. 2014; Demircioğlu et al. 2014; Bekri et al. 2006; Ravasi et al. 2012). But, there are some contradictions about the origin of hepcidin disturbance in NAFLD, mainly because there is disagreement if NAFLD or obesity are the primary cause of these changes. Changes in hepcidin levels have been observed in NAFLD with obesity (Vuppalanchi et al. 2014; Bekri et al. 2006), but also in NAFLD without obesity (Ravasi et al. 2012). Furthermore, although iron-load controls hepcidin levels in NAFLD (Siddique et al. 2014), there are studies which suggest that hepcidin disturbance can occur in spite of iron-overload (Ravasi et al. 2012; Aigner et al. 2008). It seems that the presence/absence of different regulatory factors that induce hepcidin expression are behind these discrepancies. It has to be noted that hepcidin overexpression is more consistently related to severe obesity compared to milder levels of obesity (Vuppalanchi et al. 2014; Demircioğlu et al. 2014; Bekri et al. 2006). This is important since severe obesity is not frequently observed in patients with NAFLD. Increased hepcidin expression in severe obesity originates, at least partially, from increased adipose tissue and is not under regulatory feedback control compared to liver hepcidin (Bekri et al. 2006). High levels of inflammatory cytokines seem to be the cause behind increased levels of hepcidin in severe obesity (Bekri et al. 2006). Hepcidin upregulation in NAFLD without the presence of severe obesity and iron-load is related to markers of inflammation, but also with lipid metabolism disorders (Senates et al. 2011; Ravasi et al. 2012). Mechanistic studies have shown that there exists a molecular interplay between lipid dysmetabolism and inflammatory pathways in inducing hepcidin expression in NAFLD (Lu et al. 2016).

There are also other possible culprits of increased hepcidin expression in NAFLD, such as endoplasmatic reticulum (ER) stress. ER stress is a reactive cellular response due to a disruption in ER homeostasis (Wu and Kaufman 2006). Induction of ER stress in NAFLD is believed to occur due to hepatic lipid accumulation, and is related to IR, inflammation, cellular apoptosis, which are, coincidentally, features of NAFLD and especially nonalcoholic steatohepatitis (NASH) (Malhi and Kaufman 2011). Furthermore, the expression of proteins involved in ER stress is dysregulated in NASH, while recently, the protective role of testosterone in NAFLD has been attributed to reduction of ER stress (Lee et al. 2017; Jia et al. 2017). It has to be mentioned that the impact of ER stress in the etiology of NAFLD is modified by dietary factors and obesity (Malhi and Kaufman 2011). Although the exact role of ER stress in NAFLD remains to be fully elucidated, it is increasingly evident that ER stress has an important role in the pathophysiology of NAFLD.

ER stress has been linked with iron metabolism as well. This observation is based on research that shows that downstream protein activity during ER stress (such as cyclic AMP response element-binding protein H (CREBH) and C/EBPα activity) is responsible for induction of hepcidin expression (Vecchi et al. 2009; Oliveira et al. 2009). What is more, activity of these proteins (like CREBH) stands at the crossroads between ER stress and inflammatory signaling through IL-6 (Zhang et al. 2006). Still, the direct role of ER stress in controlling hepcidin expression (in relation to other factors) in NAFLD has yet to be determined.

Low or insufficient levels of hepcidin (in relation to ferritin depos) can also have deleterious effects in the progression of NAFLD, at least in a specific subgroup of patients, especially those with comorbidities such as HH, HCV infection or ALD. In mouse models of NAFLD with hepcidin knockout there is an interesting picture of liver damage; although loss of hepcidin is associated with ameliorated liver steatosis, liver fibrosis is present early and is more pronounced compared to mice with normal hepcidin expression (Lu et al. 2016) There are studies that suggest that hepcidin levels are insufficiently increased in relation to ferritin depos in NAFLD (Mitsuyoshi et al. 2009; Barisani et al. 2008). Significant damage to the liver in NAFLD might reduce the ability of hepatocytes to increase hepcidin in relation to iron stores. Indeed, reduced hepcidin/ferritin ratio has been detected in NASH and in dysmetabolic iron-overload syndrome or DIOS (frequently present in patients with NAFLD), but not in NAFLD patients with simple steatosis (Mitsuyoshi et al. 2009; Barisani et al. 2008). One of the most striking differences between these 2 groups is the significant increase in parameters of insulin resistance (IR) in NASH (Barisani et al. 2008). IR is related to low levels of hepcidin (Le Guenno et al. 2007; Sam et al. 2013), and it could be one of the causes of insufficient hepcidin response in NASH. These observations and the data about increased levels of oxidative stress in NASH with accompanied mitochondrial structural dysfunction might mean that low hepcidin/ferritin ratio could mostly be prevalent in a subset of patients with NAFLD characterized with significant hepatocyte structural and functional damage (Sumida et al. 2009). Recently, it has been proposed that hepatocyte nuclear factor-4 alpha could be the mediator of relative hepcidin suppression in NAFLD through its effects on BMPR (Shi et al. 2017).

Iron-load is present in 1/3 of the patients with NAFLD and is a risk factor for progressive liver damage, especially when NAFLD is part of DIOS. In DIOS there is an adequate increase in hepcidin levels in response to iron-load, but the increase in hepcidin levels cannot control the rise in transferrin saturation (TS), suggesting a hepcidin-resistant state (Rametta et al. 2016). It remains to be seen how could these changes affect iron-load in the liver and the tendency towards liver fibrosis. The answer might have come from Hoki et al. study, where high levels of hepcidin in NASH were associated with iron-overload due to increased divalent metal transporter 1 (DMT1) expression in enterocytes through increased acitivity of iron-regulatory protein 1 (IRP1) (Hoki et al. 2015). IRPs are known as intracellular regulators of iron homeostasis by controlling the expression of the most important iron import and export proteins (Kühn 2015), such as DMT1. Although Hoki et al. did not evaluate hepcidin/ferritin ratio in their study, median levels of ferritin and hepcidin data from control group and patients with NASH indicate that hepcidin/ferritin ratio is lower in NASH.

In cholestasis, hepcidin levels go down, probably through suppression of interleukin 6 (IL6) induced STAT3 phophorylation by accumulated hydrophobic bile acids. Levels of hepcidin remain lower in cholestatic cirrhosis compared to non-cholestatic cirrhosis, suggesting a primary role of cholestasis in low values of hepcidin (Huang et al. 2009).

Low levels of hepcidin have been observed in patients with thalassemia (TM) as well. TM is one of the most prevalent hemoglobinopathies which is often characterized with liver fibrosis due to liver iron overload (Elalfy et al. 2013). Iron overload occurs mainly as a consequence of ineffective erythropoiesis (IE), which causes low levels of hepcidin due to increased signaling from erythrocyte precursors to liver (Kautz et al. 2015). Although transfusions recuperate hepcidin expression, IE can override regulatory control of hepcidin by iron pathways (Origa et al. 2007; Gardenghi et al. 2010). This is probably the reason why hepcidin/ferritin ratio stays low in patients with TM even after transfusions (Pasricha et al. 2013). Furthermore, the correction of hepcidin expression ameliorates iron overload in mice models with TM (Kautz et al. 2015; Gardenghi et al. 2010).

As it was mentioned previously, low hepcidin has been linked with IR. The mechanism behind this disturbance is unclear, but hepatic IR could play a role, because low levels of hepcidin are not present in diabetes mellitus (DM) type 1 (Sam et al. 2013). DM type 1 is a condition characterized by a primary immune response dysregulation in beta cells, while DM type 2 is characterized with IR in different effector tissues, including liver (Kahn et al. 2014; Atkinson et al. 2014). Studies with rodents have shown that insulin can affect hepcidin production in liver through STAT3 or possibly through other pathways as well (Wang et al. 2014). It is interesting to notice that in-vitro studies have shown that overexpression of suppressor of cytokine signaling 1 (SOCS1) protein suppresses STAT3 which results in reduced hepcidin in replicon cells infected with HCV (Miyachi et al. 2011). This would mean that in hepatic IR, defective insulin signaling could be linked with defective hepcidin expression as well. Still, the mystery behind low levels of hepcidin in hepatic IR remains unsolved, but it is important to understand the cause of this disturbance, because it might uncover a new role for insulin therapy in diabetic patients, which is the correction of hepcidin levels and iron-load as a result.

### Hepcidin role in fibrosis goes beyond control of iron-load?

Recent studies suggest that hepcidin has additional important protective features in liver fibrosis. Hepcidin can serve as a paracrine signal from hepatocytes to suppress hepatic stellate cell (HSC) activation. HSCs activation and subsequent release of profibrotic cytokines is one of the main features of liver fibrosis. By restoring hepcidin levels we can curb the process of HSC activation and subsequent liver fibrosis (Han et al. 2016). Similarly, BMP6 as one of the main inducers of hepcidin expression has been shown to have a protective role in liver fibrosis by inhibiting hepatic stellate cells activation (Arndt et al. 2015). More studies are needed to explain this new role of hepcidin in liver protection during fibrosis, but the idea is intriguing, and it might expand the importance of hepcidin in liver fibrosis.

## Low hepcidin as a biochemical marker in liver fibrosis

Though many indices of iron metabolism (ferritin, hepatic iron, TS) are frequently used diagnostic tools in detecting iron-load as a risk factor for liver fibrosis (Morrison et al. 2003; Schmitt et al. 2005; Deugnier et al. 1992), recently discovered hepcidin has gained interest because of its main function as a controller of iron efflux from cells (Nemeth et al. 2004).

In most types of HH hepcidin expression is subphysiological (Vujić 2014; Ganz et al. 2008; Kulaksiz et al. 2004) compared to ferritin (Waalen et al. 2008). In HH-HFE homozygotes hepcidin is inappropriately low even with higher levels of ferritin (van Dijk et al. 2008) (Table 1). This happens because in HH-HFE homozygotes hepcidin response to iron challenge is blunted (Sangwaiya et al. 2011). Ferritin levels are more consistently increased in another form of HH called FPN disease, which course is benign, owing to the fact that mutations with loss of function of FPN cause prevalent iron-load in macrophages compared to parenchymal cells (Zoller et al. 2005). Using ferritin values as diagnostic markers of disease severity could prove unhelpful in many cases with HH. But, using hepcidin or hepcidin/ferritin ratio can help circumvent the shortcomings of serum ferritin values. Hepcidin and hepcidin/ferritin ratio are consistently low in different types of HH (Ravasi et al. 2012; Girelli et al. 2016). Although hepcidin levels in NAFLD and DIOS are normal or high, hepcidin/ferritin ratio (compared to normal subjects) in these conditions can be low as well. Still, hepcidin/ferritin ratio is significantly lower in HH compared to NAFLD and DIOS (Ravasi et al. 2012), which means that this ratio can differentiate between HH patients and NAFLD/DIOS patients with lower hepcidin/ferritin ratios.

**Table 1:** Role of hepcidin as a biochemical marker in liver diseases

Condition	Study characteristics	Main results	References
Alcoholic cirrhosis	Prospective study (*n* = 237) Median follow-up: 68 months Serum hepcidin measurements with ELISA method	Cut-off value: < 8 μg/L Low hepcidin associated with HCC occurrence [HR = 1.76 (1.01–3.06); *P* = 0.031] Low hepcidin associated with overall death [HR = 2.84 (1.29–6.25), *P* = 0.009]	Nahon et al. 2016
ALD	*n* = 24 Serum hepcidin measurements with ELISA method	Serum hepcidin ↓ (*P* = 0.001)	Dostalikova-Cimburova et al. 2014
Chronic liver disease	*n* = 332 Liver biopsy samples and serum hepcidin measurements with mass spectrometry	Serum hepcidin ↓ (*P* < 0.0001)^a^ Hepcidin/ferritin ratio ↓ (*P* < 0.0001)^b^ Hepcidin/ferritin ratio with cut-off value < 0.1 was independently associated with liver cirrhosis [OR 5.54 (95% CI 2.49–12.35, *P* < 0.001)] Hepcidin/ferritin ratio ↓ distinguishes between F0 and F4 stages of fibrosis (AUC = 0.86)	Tan et al. 2012
Liver cirrhosis	n = 70 Serum prohepcidin measurements with ELISA method	Serum prohepcidin in all patients ↓ (*P* < 0.01) Serum prohepcidin levels ↓ in HCV and alcoholic –related cirrhosis (*P* < 0.01), but not in HBV-related cirrhosis Prohepcidin/ferritin ratio correlation with Child-Pugh score in all patients: r = 0.38, *P* = 0.01 Prohepcidin/Child-Pugh score correlation in alcohol-related liver cirrhosis: r = 0.41, *P* = 0.01	Jaroszewicz et al. 2008
Chronic hepatitis and liver cirrhosis	HCV patients (*n* = 131); HBV patients (*n* = 59) Serum hepcidin measurements with ELISA method	Serum prohepcidin ↓ in HCV patients with chronic hepatitis (P = 0.01) and liver cirrhosis (*P* = 0.037) compared to HBV patients	Nagashima et al. 2006
HCV infection	Liver biopsy from *n* = 96 patients Serum hepcidin measurements with ELISA method	Serum hepcidin ↓ (*P* < 0.001) Serum hepcidin/histological lesions correlations: necroinflammation (r = 0.259, *P* = 0.011) and fibrosis (r = 0.214, *P* = 0.036) Serum hepcidin is an independent predictor of liver cirrhosis [OR = 1.145 (1.007–1.301); *P* = 0.039]	Tsochatzis et al. 2010
HCV infection	Treatment outcomes in *n* = 31 patients Serum hepcidin measurements with mass spectrometry	Serum hepcidin ↑ (at 12 h) after treatment with pegylated IFN-α (P < 0.0001) *Correlations between hepcidin and markers of treatment response to pegylated IFN-α* I. Serum hepcidin/IFN-α: r = 0.44, *P* = 0.042 II. Serum hepcidin/IL-10: r = 0.59, *P* = 0.004	Ryan et al. 2012
HCV infection	Treatment outcomes in *n* = 15 patients Serum hepcidin measurements with mass spectrometry	Serum hepcidin ↑ (at week 1) after treatment with pegylated IFN-α (*P* = 0.013)	van Rijnsoever et al. 2016
HCV infection	Treatment outcomes in *n* = 73 patients Serum hepcidin measurements with mass spectrometry	Hepcidin/ferritin ratio ↓ (*P* = 0.028) Serum hepcidin ↑ in patients with SVR after 48 weeks of treatment (*P* < 0.01) Hepcidin/ferritin ratio ↑ in patients with SVR after 48 weeks of treatment (*P* < 0.01)	Fujita et al. 2008
HCV infection	Treatment outcomes Retrospective study (*n* = 50) Serum hepcidin measurements with mass spectrometry	Serum hepcidin ↓ in patients with SVR (but did not reach statistical significance)	Kohjima et al. 2015
HCV infection	*n* = 81 Serum hepcidin measurements with ELISA method	Serum hepcidin ↓ (*P* < 0.001) Serum hepcidin/ferritin(quartiles) ↓ (*P* < 0.001)	Girelli et al. 2009
HCV infection	n = 9 Serum hepcidin measurements with mass spectrometry	Hepcidin/ferritin ratio ↓ (*P* = 0.0068) Hepcidin/ferritin ratio ↓ after phlebotomy (*P* = 0.0338)	Sugimoto et al. 2009
Chronic liver disease	*n* = 34 (children) Liver biopsy Serum hepcidin measurements with ELISA method	Hepcidin/ferritin ratio ↓ in patients with Child-Pugh score B + C compared to Child-Pugh score A (*P* = 0.03) Hepcidin/ferritin ratio ↓ in patients with severe fibrosis vs no fibrosis/mild fibrosis (*P* < 0.05)	Cakir et al. 2015
HBV-related cirrhosis	*n* = 70 Serum hepcidin measurements with ELISA method	Serum hepcidin ↓ (*P* < 0.001) Serum hepcidin/TS ratio ↓	Lin et al. 2013
Chronic HBV infection	*n* = 46 Nanopore film-based assay	Serum hepcidin ↓ in cirrhotic HBV infection compared to non-cirrhotic HBV infection (*P* < 0,05) Serum hepcidin ↓ in Child-Pugh class C compared to Child-Pugh class A (but without reaching statistical significance)	Wang et al. 2016
NAFLD	*n* = 51 Serum hepcidin measurements with ELISA method	Hepcidin correlates with hepatic lipid content (r = 0.42, *P* = 0.0024) Hepcidin ↑ in NASH vs non-NASH NAFLD (*P* = 0.01) Hepcidin differentiates between early and later stages of liver fibrosis (*P* < 0.0001) Hepcidin is an independent predictor of liver fibrosis [OR = 1.03 (1.00-1.05); *P* = 0.022]	Ryan et al. 2017
NAFLD	*n* = 54 Serum hepcidin measurements with ELISA method	Hepcidin is an independent predictor of advanced liver fibrosis [OR = 560.72 (5.98-5255.33); *P* = 0.006] Hepcidin cut-off value of 45.00 ng/mL differentiates between simple steatosis and NASH	Jamali et al. 2016
DIOS	*n* = 18 Serum hepcidin measurements with ELISA method	Hepcidin resistance index^c^ ↑(*P* = 0.0002)	Rametta et al. 2016
NAFLD, DIOS, HH-HFE, THAL	n = 15 (NAFLD), *n* = 47 (DIOS), n = 23 (HH-HFE), *n* = 9 (THAL) Serum hepcidin measurements with mass spectrometry	Serum hepcidin ↓ in HH-HFE vs controls (*P* < 0.01) Serum hepcidin ↓ in HH-HFE vs DIOS (*P* < 0.01) Hepcidin/ferritin ratio ↓ in DIOS vs controls (*P* < 0.01) Hepcidin/ferritin ratio ↓ in HFE-HH vs controls (*P* < 0.01) Hepcidin/ferritin ratio ↓ in THAL vs controls (*P* < 0.01) Hepcidin/ferritin ratio ↓ in HFE-HH vs NAFLD (*P* < 0.01) Hepcidin/ferritin ratio ↓ in HFE-HH vs DIOS (*P* < 0.01)	Ravasi et al. 2012
Liver autoimmune diseases vs NAFLD and HBV/HCV infections	AICD (n = 34) AIH (*n* = 16) NAFLD (*n* = 32) HBV infection (*n* = 23) HCV infection (*n* = 21) Serum hepcidin measurements with ELISA method	Serum hepcidin ↓ in AIH compared to NAFLD (*P* < 0.001), HBV infection (*P* < 0.001), HCV infection (*P* = 0.001) Serum hepcidin/ferritin ratio ↓ in AIH compared to NAFLD (*P* < 0.001), HBV infection (*P* < 0.001), HCV infection (*P* < 0.001) Serum hepcidin ↓ in AICD compared to NAFLD (*P* < 0.001), HBV infection (*P* < 0.001), HCV infection (*P* < 0.001) Serum hepcidin/ferritin ratio ↓ in AICD compared to NAFLD (*P* < 0.001), HBV infection (*P* < 0.001), HCV infection (*P* < 0.001) Serum hepcidin ↓ in HCV infection compared to HBV infection (*P* = 0.018)	Lyberopoulou et al. 2015
Biliary atresia	*n* = 10 (early stage disease); n = 9 (late stage disease) Liver biopsy samples Serum hepcidin measurements with ELISA method	Hepcidin mRNA ↓ in late stage disease (cirrhosis) compared to early stage disease (*P* < 0.001) Serum hepcidin ↓ in late stage disease (cirrhosis) compared to early stage disease (*P* = 0.02)	Huang et al. 2009
HH	n = 5 (HH-HFE), *n* = 6 (HH-HJV) Serum hepcidin measurements with ELISA method	Hepcidin/ferritin ratio ↓ in untreated HH-HFE Serum hepcidin ↓ in HH-HJV (*P* < 0.001)	Ganz et al. 2008
Iron-overload conditions	n = 13 Serum hepcidin measurements with mass spectrometry	Serum hepcidin ↓ in HH-HFE, HH-HJV, HH-TFR2 vs serum hepcidin ↑ in FPN disease (*P* < 0.01)	Kaneko et al. 2010
HFE-HH (C282Y)	*n* = 22 Serum hepcidin measurements with mass spectrometry	Serum hepcidin ↓ in untreated homozygotes (*P* < 0.01)^d^ Hepcidin/ferritin ratio ↓ in untreated homozygotes (*P* < 0.001)	van Dijk et al. 2008
HFE-HH (C282Y)	n = 9 Serum hepcidin measurements with ELISA method	Serum hepcidin ↓ in untreated homozygotes (*P* = 0.0002) Hepcidin response to oral iron challenge ↓ (AUC: *P* = 0.0127)	Sangwaiya et al. 2011
FPN disease type A	n = 8 Serum prohepcidin measurements with ELISA method	Serum prohepcidin ↑ in untreated patients compared to normal control and treated patients with FPN disease	Zoller et al. 2005

*Abbreviations*: *AICD* autoimmune cholestatic disease, *AIH* autoimmune hepatitis, *ALD* alcoholic liver disease, *AUC* area under curve, *DIOS* dysmetabolic iron overload syndrome, *ELISA* enzyme-linked immunosorbent assay, *FPN* ferroportin, *HBV* hepatitis B virus, *HCC* hepatocellular carcinoma, *HCV* hepatitis C virus, *HFE* hemochromatosis protein, *HH* hemochromatosis, *HJV* hemojuvelin, *HR* hazard ratio, *IFN* interferon, *NAFLD* nonalcoholic fatty liver disease, *NASH* nonalcoholic steatohepatitis, *OR* odds ratio, *SVR* sustained virological response, *TFR2* transferrin receptor 2, *THAL* thalassemia, *TS* transferrin saturation

Up (↑) and down (↓) arrows are presented to signify changes in levels of biochemical markers or gene expression; down arrow (↓) means that a specific biochemical marker or genetic expression levels are low, while up arrow (↑) means that a specific biochemical marker or genetic expression levels are high

^a^Significance was observed between patients with liver conditions (ALD, AIH, HBV infection, HCV infection, NAFLD, PBC, PSC) and disease-control subjects (non-liver rheumatological, renal and hematological disease)

^b^Significance was observed between patients with liver conditions (ALD, AIH, HBV infection, HCV infection, NAFLD, PBC, PSC) and disease-control subjects (non-liver rheumatological, renal and hematological disease) and healthy controls

^c^Hepcidin resistance index is defined as the ability of hepcidin spike to control the rise in TS

^d^In HH-HFE homozygotes with high ferritin levels levels of hepcidin were lower than controls, but did not reach statistical significance

While levels of hepcidin in HH are mostly inadequate, in FPN disease they are normal to high, which makes serum hepcidin a helpful biochemical marker in differential diagnosis of HH (Zoller et al. 2005; Kaneko et al. 2010; Papanikolaou et al. 2005; Sham et al. 2009). Making the case for serum hepcidin in FPN disease is also the fact that it can help in differential diagnosing between 2 forms of FPN disease in conjunction with ferritin and TS levels (Zoller et al. 2005). But, using hepcidin as a biomarker in HH is not always helpful; for example, in patients with HH-HFE and co-existent inflammatory condition, acute bouts of increased inflammatory activity increase levels of hepcidin and hepcidin/ferritin ratio and may mask the condition (van Deuren et al. 2009). It has to be mentioned that in more severe forms of HH like HH-TFR2, hepcidin levels rise during inflammation but hepcidin/ferritin ratio is mostly low (Nemeth et al. 2005). This means that inflammatory activity cannot mask hepcidin/ferritin ratio in all cases with HH. In addition, iron challenge in HH-TFR2 does note elicit a hepcidin response, while in homozygotes with HH-HFE the response is present, albeit insufficient for the level of iron-load (Girelli et al. 2011). Hepcidin levels can be beneficial in predicting the need for phlebotomies as well (Girelli et al. 2016), because higher levels of hepcidin will reduce the iron-load in cells by suppressing iron release form macrophages and enterocytes.

The value of serum ferritin as a marker of liver fibrosis is limited in other diseases as well. In NAFLD ferritin levels as a marker of liver fibrosis has a poor sensitivity value of 16%-41% (Angulo et al. 2014). Serum ferritin often increases as a consequence of inflammation, which may be the reason behind low sensitivity value of iron depos (ferritin) in iron-overload states (Kell and Pretorius 2014).

In clinical practice the use of aspartate aminotransferase (AST) levels and platelet count has been proposed to have a 100% negative predictive value for high degree fibrosis (Castiella et al. 2008). But, silent liver fibrosis in HH (characterized by normal transaminase levels) has been detected in up to 18% of patients, which means that transaminase values can underestimate detection of liver injury during iron overload (Beaton and Adams 2008).

Low levels of hepcidin with cut-off value of < 8 μg/L are an independent predictor of mortality and hepatocellular carcinoma (HCC) in alcoholic cirrhosis (Nahon et al. 2016; Dostalikova-Cimburova et al. 2014). Association of hepcidin with cirrhosis is in terms with experimental studies where iron supplementation drastically exacerbates alcohol-induced liver fibrosis (Tsukamoto et al. 1995). Similarly, in HBV cirrhotic patients levels of hepcidin are low compared to non-cirrhotic HBV patients (Wang et al. 2016; Yonal et al. 2010). Studies suggest that these values do not show changes when comparison is made with healthy controls (Wang et al. 2016; Jaroszewicz et al. 2008), although not all authors agree (Lin et al. 2013; Yonal et al. 2010). In any case, levels of hepcidin in HCV-related cirrhosis and alcoholic-related cirrhosis are consistently and significantly lower than in HBV-related cirrhosis, which indicates a disease-specific factor that affects hepcidin levels (Nagashima et al. 2006; Tan et al. 2012; Jaroszewicz et al. 2008).

In chronic HCV infection levels of hepcidin and hepcidin/ferritin ratio are low, even without the presence of liver cirrhosis (Nagashima et al. 2006; Girelli et al. 2009; Tsochatzis et al. 2010; Fujita et al. 2008). But in Sugimoto et al. study, levels of hepcidin were higher in chronic HCV patients, although this might have occurred because of higher levels of inflammation (Sugimoto et al. 2009). In any case, even in this study, hepcidin/ferritin ratio was low and stayed low even after phlebotomy. In HCV infection, hepcidin can serve as a biomarker of treatment outcome as well. Treatment with pegylated interferon increases serum hepcidin, and this increase is correlated with parameters of treatment response (Ryan et al. 2012). The correction of hepcidin and hepcidin/ferritin levels persists with duration of therapy, even after 48 weeks of treatment (Fujita et al. 2008; van Rijnsoever et al. 2016; Strnad et al. 2014). These results are characteristic of patients with sustained virological response (SVR), which is defined as an aviremic status persisting for 24 weeks after antiviral therapy. On the other hand, Jaroszewicz et al. and Kohjima et al. studies yielded opposite results compared to former studies (Jaroszewicz et al. 2010; Kohjima et al. 2015). It has to be mentioned that Jaroszewicz et al. have used prohepcidin as a biomarker, which is a precursor to hepcidin, which does not always correlate with hepcidin levels (Valore and Ganz, 2008). Jaroszewicz et al. have proposed that HCV might interfere with the process of converting prohepcidin to its mature form, which remains to be resolved. On the other hand, a careful examination of patient results in Kohjima et al. study indicates a presence of different levels of iron-load between patients; levels of hepcidin in patients with SVR although lower than in controls, did not correlate negatively with FPN expression. This indicates that the significant increase in FPN expression in these patients was due to increased iron-load. In non-SVR patients hepcidin expression was higher compared to FPN. The contradiction from this study might have been solved if authors had used hepcidin/ferritin ratio in their statistical analysis, although in Kohjima et al. study expression levels of ferritin were higher compared to hepcidin levels in HCV patients, suggesting a low hepcidin/ferritin ratio.

Although levels of serum hepcidin are not suppressed in NAFLD/NASH, at least not in early stages of the disease, hepcidin eventually starts to drop in NAFLD with advanced fibrosis, similar to other liver diseases (Jamali et al. 2016). Furthermore, serum hepcidin can serve as an independent marker of fibrosis stage and severity of fibrosis in NAFLD (Ryan et al. 2017; Jamali et al. 2016).

When hepcidin values are corrected by iron-load (by using hepcidin/ferritin ratio), they show low values in patients with severe liver fibrosis (Cakir et al. 2015). What is more important this ratio can differentiate between advanced fibrosis and lack of fibrosis (Tan et al. 2012). Furthermore, hepcidin/ferritin ratio with cut-off value of < 0.1 is independently associated with liver cirrhosis (Tan et al. 2012). Tan et al. study has shown that hepcidin/ferritin ratio, is lower in cirrhotic than non-cirrhotic patients with HBV, HCV and NAFLD (Sun et al. 2015). But, in ALD, hepcidin/ferritin ratio was low irrespective of cirrhosis. This shows that suppression of hepcidin in ALD is directly related with effects of alcohol on hepcidin expression. Using hepcidin/ferritin ratio could be useful when ferritin changes are not caused by iron-load (i.e. inflammation). On the other hand, serum ferritin levels cannot be used as a biochemical marker that differentiates between stages of fibrosis (Angulo et al. 2014). By using hepcidin/ferritin ratio we could circumvent the low sensitivity value of ferritin levels in this setting. In NAFLD hepcidin/ferritin ratio could be of value in detecting a subset of patients in whom liver damage could progress to liver fibrosis, though this remains to be examined by future studies.

Low values of hepcidin have been observed in AILD (Lyberopoulou et al. 2015). It is interesting to notice that AILD patients have significantly lower values of hepcidin compared to HCV patients. Also, long-term treatment in AILD does not affect hepcidin levels (Lyberopoulou et al. 2015). These data show that low values of hepcidin seem to be related intrinsically with the pathogenic mechanisms behind AILD, but also they can be used as a simple biochemical parameter in diagnosing patients with high suspicion of AILD.

It is clear that hepcidin can serve as an important biochemical parameter in liver fibrosis. Hepcidin/ferritin ratio could improve hepcidin sensitivity because it might detect early fibrosis. Unfortunately, hepcidin/ferritin ratio has not been compared to other markers of liver fibrosis, which would create a clearer picture of the importance of this ratio in liver fibrosis.

## Conclusion

This review has examined the importance of low levels of hepcidin in liver fibrosis. The main mechanisms of this disturbance are realized through alcohol-induced injury and to a lesser extent by viral infection with HCV, while the role of HBV in this setting is secondary to dramatic liver damage seen in cirrhosis caused by HBV. Other mechanisms of low hepcidin include unknown autoimmune dysregulation, cholestasis and hepatic IR. In NAFLD insufficient hepcidin production in response to iron-load seems to be related with more prominent liver damage, though this remains to be confirmed along with detailed mechanistic explanations behind these changes. Low levels of hepcidin can cause iron-overload, but as recent data suggest, low hepcidin can have additional repercussion to liver architecture because of hepcidin ability to control HSC activation, which is one of the main pathophysiological features in liver fibrosis. These mechanisms are in concert with clinical studies that have established hepcidin and hepcidin/ferritin ratio as an important biochemical parameter of liver fibrosis with the ability to predict patient mortality and increased risk of HCC. Still, more comprehensive studies are needed to discover the real role of hepcidin in relation to standard biochemical markers of liver fibrosis.

Recovering hepcidin levels might curb the process of liver fibrosis. In models of mice with liver fibrosis, hepcidin overexpression with ad-hepcidin attenuates liver fibrosis, which is accompanied with correction of the values for AST, ALT and lactate dehydrogenase (LDH) (Han et al. 2016). It is interesting to notice that similar liver antifibrotic actions have been observed with BMP6 overexpression as well (Arndt et al. 2015). The strategy to recuperate hepcidin signaling in clinical practice could include the use of synthetic hepcidins or of already established drugs. Mini-hepcidins and other synthetic prohepcidin drugs can reduce iron-overload by correcting hepcidin levels, and thus ameliorate liver fibrosis (Ramos et al. 2012; Schmidt et al. 2015). On the other hand, it is still not clear if iron-depletion might be beneficial in one of the most prevalent liver condition such as NAFLD. Some smaller studies have associated phlebotomy with improvements in hepatic functional parameters (Valenti et al. 2007; Valenti et al. 2014; Sumida et al. 2006), but in other studies the benefit of phlebotomy in NAFLD was small or nonexistent (Adams et al. 2015; Murali et al. 2017; Beaton et al. 2013). While the debate continues (Garg et al. 2013; Ryan et al. 2015), some national guidelines are embracing the possibilities of phlebotomy in NAFLD, while cautioning that any official recommendation about the use of phlebotomy should be taken into account only after we obtain confident results from large and long-term trials (Watanabe et al. 2015). The biggest question to be resolved by these trials will be to find out that if phlebotomy is beneficial in NAFLD, then, is this benefit reserved to a subset of patients or to a much larger group of patients.

The use of synthetic hepcidins is not without caution, because it can cause side effects, such as anemia (Ramos et al. 2012). The new synthetic hepcidin named LJPC-401 has shown early promise in phase 1 clinical trial, with less adverse effects compared to its earlier counterparts. Later in 2017, this new drug will be used in a randomized multi-center study in beta thalassemia patients (La Jolla Pharmaceutical, 2016). This study was approved by European Medicines Agency and it will unveil the treatment potential of LJPC-401 in this debilitating blood disorder which is accompanied with liver damage (Elalfy et al. 2013). Calcium channel blockers (CCBs), on the other hand, reduce iron-load in liver and reverse hepatic fibrosis by mechanisms that include lowering of ferritin and DMT1 levels, but there are no studies that have examined if CCBs affect hepcidin mode of production or action (Zhang et al. 2016). These therapeutic options could prove important, because they can circumvent potential toxic effects of chelator therapy or secondary inhibition of hepcidin production due to phlebotomy (Pak et al. 2006; Porter and Huehns, 1989).

## Abbreviations

AILD: Autoimmune liver disease
ALD: alcoholic liver disease
AST: Aspartate aminotransferase
BMP6: Bone morphogenetic protein 6
BMPR: BMP receptor
C/EBP: CCAAT-enhancer-binding protein
CCB: Calcium channel blocker
CRAMP: Cathelin-related antimicrobial peptide
CREBH: Cyclic AMP response element-binding protein H
DIOS: Dysmetabolic iron-overload syndrome
DM: Diabetes mellitus
DMT1: Divalent metal transporter 1
ER: Endoplasmatic reticulum
FPN: Ferroportin
*HAMP*: Hepcidin antimicrobial peptide
HBV: Hepatitis B virus
HCC: Hepatocellular carcinoma
HCV: Hepatitis C virus
HH: Hemochromatosis
HJV: Hemojuvelin
HSC: Hepatic stellate cell
IE: Ineffective erythropoiesis
IL-6: Interleukin-6
IR: Insulin resistance
IRP: Iron regulatory protein
JAK2/STAT3: Janus kinase 2/signal transducer and activator of transcription 3
LDH: Lactate dehydrogenase
LPS: Lipopolysaccharide
LSECs: Liver sinusoidal endothelial cells
NAFLD: Non-alcoholic fatty liver disease
NASH: Nonalcoholic steatohepatitis
SMAD: s-mothers against decapentaplegic
SOCS1: Suppressor of cytokine signaling 1
SVR: Sustained virological response
TFR2: Transferrin receptor 2
TLR4: Toll-like receptor 4
TM: Thalassemia
TS: Transferrin saturation
